# Supplementary material for: Antibodies blocking PlGF or VEGF interactions with the NRP1 receptor mediate antiproliferative effects
Source: J Biol Chem. 2026 Apr 17;302(6):111476. doi: 10.1016/j.jbc.2026.111476 (PMC13208732; doi:10.1016/j.jbc.2026.111476)
Supplement: Figures S1–S20 [file mmc1.pdf]

Supporting information for:

Antibodies blocking PlGF or VEGF interactions with the NRP1 receptor mediate anti-proliferative effects

Samuel A. Blackman[a], Ahlam N. Qerqez[a], Alison G. Lee[b], Nicole V. Johnson[b], Justin M. Owens[a], Grace S. Lai[a], Emma C. Aldrich[c], Kayla G. Sprenger[c], Jangsoon Lee[d], Sophie Verrier,[e] Martin Stoddart,[e] Annalee W. Nguyen[a], Jennifer A. Maynard[a]

[a]McKetta Department of Chemical Engineering
University of Texas at Austin
200 E Dean Keeton St
Austin, TX 78712

[b]Interdisciplinary Life Sciences Graduate Program
The University of Texas at Austin
North Hackerman Building
100 East 24th St, NHB 4500
Austin, TX 78712

[c]Department of Chemical and Biological Engineering
University of Colorado Boulder
3415 Colorado Ave
Boulder, CO 80309, USA

[d]University of Hawaii Comprehensive Cancer Center
University of Hawaii
701 Ilao St
Honolulu, HI 96813, USA

[e]AO Research Institute Davos
Clavadelerstrasse 8,
7270 Davos Platz, Switzerland

Supplementary methods: qPCR

To validate HUVEC gene expression in tube formation assay on Matrigel, we completed quantitative gene expression for VEGF, PlGF, NRP1, VEGFR1, and VEGFR2. HUVECs were seeded 5000 cell/well on Matrigel and let incubate for 16-18 h, just like the tube formation assay. 16 wells per replicate were combined for adequate RNA collection. RNA was then isolated by RNeasy mini kit (Qiagen) and converted to cDNA by SuperScript VILO (ThermoFisher). The following primer/probe TaqMan^{TM} Assays (ThermoFisher) were used for gene identification on a QuantStudio^{TM} Pro6 (ThermoFisher): VEGFA (HS00900055_m1), PlGF (HS00182176), NRP1 (HS00826128_m1), VEGFR1 (HS00176573_m1), and VEGFR2 (HS00176676_m1).Relative gene expression was calculated by the following formula, assuming 100% primer efficiency, where the E_{ref} was the house keeping gene RPLP0 (MicroSynth):

$$RE = \frac{\left(E_{Ref}\right)^{Ct_{Ref}}}{\left(E_{GOI}\right)^{Ct_{GOI}}}$$

A, Human PLGF1 vs PlGF2

```
huPlGF1         LPAVPPQQWALSAGNGSSEVEVVPFQEVWGRSYCRALERLVDVVSEYPSEVEHMFSPSCV    60
huPlGF2         LPAVPPQQWALSAGNGSSEVEVVPFQEVWGRSYCRALERLVDVVSEYPSEVEHMFSPSCV    60
                ************************************************************

huPlGF1         SLLRCTGCCGDENLHCVPVETANVTMQLLKIRSGDRPSYVELTFSQHVRCECRPLREKMK    120
huPlGF2         SLLRCTGCCGDENLHCVPVETANVTMQLLKIRSGDRPSYVELTFSQHVRCECRPLREKMK    120
                ************************************************************

huPlGF1         PER---------------------CGDAVPRR    131
huPlGF2         PERRRPKGRGKRRREKQRPTDCHLCGDAVPRR    152
```

B, Human PlGF2 vs $VEGFA_{165}$

```
VEGF-165        --------APMAEGGGQNHHEVVKFMDVYQRSYCHPIETLVDIFQEYPDEIEYIFKPSCV   52
huPlGF2         LPAVPPQQWALSAGNGSSEVEVVPFQEVWGRSYCRALERLVDVVSEYPSEVEHMFSPSCV   60
                          :: *.*... *** * :*: ****: :* ***:..***.*:*::*.****

VEGF-165        PLMRCGGCCNDEGLECVPTEESNITMQIMRIKPHQGQHIGEMSFLQHNKCECRPKKDRAR   112
huPlGF2         SLLRCTGCCGDENLHCVPVETANVTMQLLKIRSGDRPSYVELTFSQHVRCECRPLREKMK   120
                 *:** ***.**.*.***.* :*:***:::*:  :     *::* ** :***** ::: :

VEGF-165        QEN--PCGPCSERRKHLFVQDPQTCKCSCKNTDSRCKARQLELNERTCRCDKPRR        165
huPlGF2         PERRRPKGRGKRRRE---KQRPTDCHLCGD--------------------AVPRR        152
                 *.  * *  ..**:    * *  *: . .                      ***
```

C, Human PlGF2 vs mouse PlGF2

```
huPlGF2         LPAVPPQQWALSAGNGSSEVEVVPFQEVWGRSYCRALERLVDVVSEYPSEVEHMFSPSCV    60
mPlGF2          ----VHSQGALSAGNNSTEVEVVPFNEVWGRSYCRPMEKLVYILDEYPDEVSHIFSPSCV    56
                       .* ******.*:*******:********* :*:** ::.***.**.*:******

huPlGF2         SLLRCTGCCGDENLHCVPVETANVTMQLLKIRSGDRP-SYVELTFSQHVRCECRPLREKM    119
mPlGF2          LLSRCSGCCGDEGLHCVPIKTANITMQILKIPPNRDPHFYVEMTFSQDVLCECRPILETT    116
                  * **:******.*****::***:***:***  .  *  ***:****.* *****: *.

huPlGF2         KPERRRPKGRGKRRREKQRPTDCHLCGDAVPRR  152
mPlGF2          KAERRKTKGKRKRSRNSQTEEPHP---------  140
                 * ***: **: ** *:.*
```

D, Human $VEGFA_{121}$ vs $VEGFA_{165}$

```
VEGF-121        APMAEGGGQNHHEVVKFMDVYQRSYCHPIETLVDIFQEYPDEIEYIFKPSCVPLMRCGGC   60
VEGF165         APMAEGGGQNHHEVVKFMDVYQRSYCHPIETLVDIFQEYPDEIEYIFKPSCVPLMRCGGC   60
                 ************************************************************

VEGF-121        CNDEGLECVPTEESNITMQIMRIKPHQGQHIGEMSFLQHNKCECRPKKDRARQEK-----   120
VEGF165         CNDEGLECVPTEESNITMQIMRIKPHQGQHIGEMSFLQHNKCECRPKKDRARQENPCGPC   120
                 ******************************************************:

VEGF-121        ---------------------------------------CDKPRR        121
VEGF165         SERRKHLFVQDPQTCKCSCKNTDSRCKARQLELNERTCRCDKPRR        165
```

Figure S1. Sequence alignments of growth factor isoforms. (**A**), Human PlGF1 (Uniprot P49763-2) versus human PlGF2 (Uniprot P49763-3). The PlGF2 exon 6 insert that encodes the heparin binding domain is highlighted in yellow, the heparin binding motifs (BBXB) are underlined, the c-terminal NRP1-binding peptide highlighted in blue and cysteines in bold. (**B**), Comparison of the 42% identical human PlGF2 versus $VEGFA_{165}$ (Uniprot P15692-4) sequences, with conserved cysteines in bold and putative or confirmed heparin binding sites underlined (R123, R124, R159 for VEGF). **C,** Comparison of the 60% identical human PlGF2 vs mouse PlGF2 (Uniprot # Q544A5), **D,** $VEGFA_{165}$ versus $VEGFA_{121}$ (Uniprot P15692-9) showing the exon 7 and 8a sequences responsible for heparin- and NRP1-binding, respectively. Alignments performed with Clustal Omega.

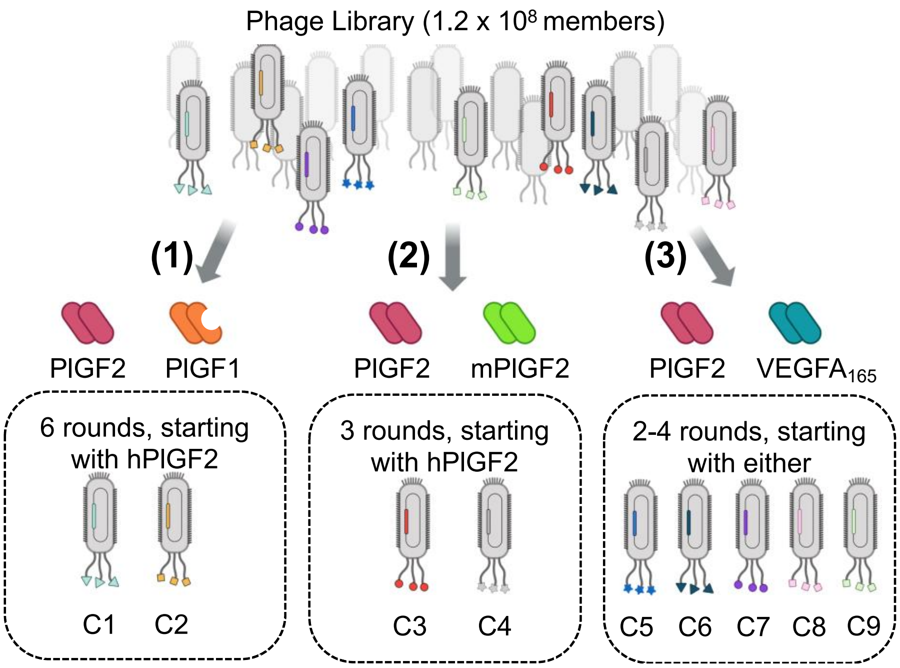

Figure S2. Phage panning strategies. A phage library was generated from mice immunized with hPlGF2 and panned using three strategies: **(1)** hPlGF2 with screening to identify clones not binding PLGF1, **(2)** alternate panning against mPlGF2 and hPlGF2, and **(3)** alternate panning against $hVEGFA_{165}$ and hPlGF2 to yield the indicated clones.

A.

Clone	V_H germline chain usage			V_H somatic mutations	V_L germline chain usage		V_L somatic mutations
C1	IGHV14-4	IGHD2-4	IGHJ4	2	IGKV6-15	IGKJ2	5
C2	IGHV1-9	IGHD1-1	IGHJ3	16	IGKV6-15	IGKJ5	9
C3	IGHV1-47	IGHD6-1	IGHJ4	10	IGKV6-17	IGKJ1	2
C4	IGHV1-9	IGHD2-4	IGHJ2	15	IGKV4-57	IGKJ5	6
C5	IGHV1-47	IGHD2-2	IGHJ4	6	IGKV12-44	IGKJ5	4
C6	IGHV14-4	IGHD4-1	IGHJ4	6	IGKV3-4	IGKJ1	4
C7	IGHV14-4	IGHD1-1	IGHJ2	3	IGKV6-15	IGKJ2	2
C8	IGHV1-9	IGHD2-14	IGHJ4	2	IGKV14-126	IGKJ5	4
C9	IGHV1-18	IGHD6-10	IGHJ4	5	IGKV3-7	IGKJ2	3

B.

>G6-31 VH
EVQLVESGGGLVQPGGSLRLSCAASG**FTISDYWIH**WVRQAPGKGLEWVAG**ITPAGGYTYYADSVKG**RFTISADTSKN
TAYLQMNSLRAEDTAVYYCAR**FVFFLPYAMDY**WGQGTLV

>G6-31 VL

DIQMTQSPSSLSASVGDRVTITC**RASQDVSTAVA**WYQQKPGKAPKLLIY**SASFLYS**GVPSRFSGSGSGTDFTLTISSLQPE
DFATYYC**QQGYGNP**FTFGQGTKVEIKR

>C9.V2 VH
EVQLVESGGGLVQPGGSLRLSCAASG**FTFTNTWIS**WVRQAPGKGLEWVGS**ITPANGYTNYADSVKG**RFTISADTSKN
TAYLQMNSLRAEDTAVYYCAR**AVYPWFFAY**WGQGTLVTVS

>C9.V2 VL

DIQMTQSPSSLSASVGDRVTITC**RASQYVSHAVA**WYQQKPGKAPKLLIY**SASFLYS**GVPSRFSGSGSGTDFTLTISSLQP
EDFATYYC**QSAYTPPTT**FGQGTKVEIKR

>C1 VH
EVQLQQSGAELVRSGASVKLSCTASG**FNIKDYYMH**WVKQRPEQGLEWIGW**IDPEDGDTEYAPKFQG**KATMTADTSS
NTAYLQLSSLTSEDTAVYYCNA**PDDDYDSSYAMDY**WGQGTSVTVS

>C1 VL

DIVMTQSQKFMSTSVGDRVSVTC**KASQNVGTNVA**WYQQKPGQSPKALIY**SASYRYS**GVPDRFTGSGSGTDFTLTISNV
QSEDLAEYIC**QQYDTFPYT**FGGGTKLEIKR

>C2 VH

QVQLQQSGAELMKPGASVKISCKAPG**YSFTSYWIE**WVKQRPGHGLEWIGE**ILPGSISPNYNEQFRG**KATITADISSNT
AYMQLSSLTSEDSAVYYCAR**ERAVYGSKFAY**WGQGTLVTVS

>C2 VL

DIVMTQSHKFMSASVGDRVSITC**KASQDVGTAVA**WYQQKPGQSPKALIY**SASYRYS**GVPDRFTGSGSGTDFTLTISNVQ
SEDLAEYFC**QQYNSYPLT**FGAGTKLELKR

>C3 VH

QVQLQQPGAELVRPGASVKLSCKASG**YTFTSYWIN**WVKQRPGQGLEWIGN**IYPSDSYTNYNQKFRD**KATLTVDKSSS TAYMQLSRPTSEDSAVYYCSR**WDAPYAMDY**WGQGTSVTVS

>C3 VL

DIVMTQSHKFMSTSVGDRVSITC**KASQDVSTAVA**WYQQKPGQSPKLLIY**SASYRYT**GVPDRFTGSGSGTDFTFTISSVQ AEDLAVYYC**QQHYSTPWT**FGGGTKLEIKR

>C4 VH

EVQLQQSGAELARPGASVKMSCKATG**YTFSIYWIE**WVKQRPGHGLEWIGE**ILPGSGSTNYNEKFKG**KATFTADTSSNT AYMQLSSLTSEDSAVYYCAR**WPIMIAGHYLDY**WGQGTTLTVSS

>C4 VL

DIVMTQSPAIMSASPGEKVTITC**SASSSVSYMH**WFQQKPGTSPKLWIY**STSNLAS**GVPARFSGSGSGTSYSLTISRMEAE DAATYYC**QQRSSYPLT**FGAGTKLEIKR

>C5 VH

QVQLQQSGAELVRPGASVKLSCKASG**YTFTSYWIN**WVKQRPGQGLEWIGN**IYPSDGYTNYNQKFKD**KATLTVDKSTS TAYMQLSSPTSEDSAVYYCTR**WYYAYGGGFAMDY**WGQGTSVTVS

>C5 VL

DIVMTQSPASLSASVGETVTITC**RASENIYSYLA**WYQQKQGKSPQLLVY**NAKTLAE**GVPSRFSGSGSGTQFSLKINSLQP EDFGSYYC**QHHYGTPLT**FGAGTKLELKR

>C6 VH

DVKLRESGAELVRSGASVKLSCTASG**FNIKDYYMH**WVKQRPEQGLEWIGW**IDPENGDTEYAPKFQG**KATMTADTSSN TAYLQLSSLTSEDTAVYYCNA**WGAMDY**WGQGTSVTVS

>C6 VL

DIVMTQSPASLAVSLGQRATISC**KASQSVDYDGDSYMN**WYQQKPGQPPKLLIY**AASNLES**GIPARFSGSGSGTDFTLNI HPVEEEDAATYYC**QQSYEDPWT**FGGGTKLEIKR

>C7 VH

QVQLQQSGAELVRSGASVKLSCTASG**FNIKDYYMH**WVKQRPEQGLEWIGW**IDPENGDTEYAPKFQG**KATMTADTSS NTAYLQLSSLTSEDTAVYYCNA**WYYGSSYNYFDY**WGQGTTLTVS

>C7 VL

DIVMTQAQKFMSTSVGDRVSVTC**KASQNVGTNVA**WYQQKPGQSPKALIY**SASYRYS**GVPDRFTGSGSGTDFTLTNSN VQSEDLAEYFC**QQYNSYPPT**FGGGTKLEIKR

>C8 VH

QVQLQQSGAELMKPGASVKISCKATG**YTFSSYWIE**WVKQRPGHGLEWIGE**ILPGSGSTNYNEKFKG**KATFTADTSSNT AYMQLSSLTSEDSAVYYCAR**SYRYDDYAMDY**WGQGTSVTVS

>C8 VL

DIQMTQSPSSMYASLGERVTITC**KASQDIKSYLS**WYQQKPWKSPKTLIY**YATSLAD**GVPSRFSGSGSGQDYSLTISSLES DDTATYYC**LQHGESPPT**FGAGTKLEIKR

>C9 VH

QVQLQQSGAELVRPGASVTLSCKASG**YTFTDYEMH**WAKQTPVHGLEWIGA**IDPETGGTAYNQNFKG**KATLTADKSSST AYMELRSLTSEDSAVYYCAR**SYDYSAMDY**WGQGTSVTVS

>C9 VL

DIVMTQSPASLAVSLGQRATISC**RASQSVSTSSYSYMH**WYQQKPGQPPKLLIK**YASNLES**GAPARFSGSGSGTDFTLNI HPVEEEDTATYYC**QHSWEIPYT**FGGGAKLEIKR

Figure S3. Antibody heavy and light chain sequences. (**A**), germline usage for each antibody. (**B**), Sequences for the antibody variable region used in this work, with Kabat-defined CDRs underlined.

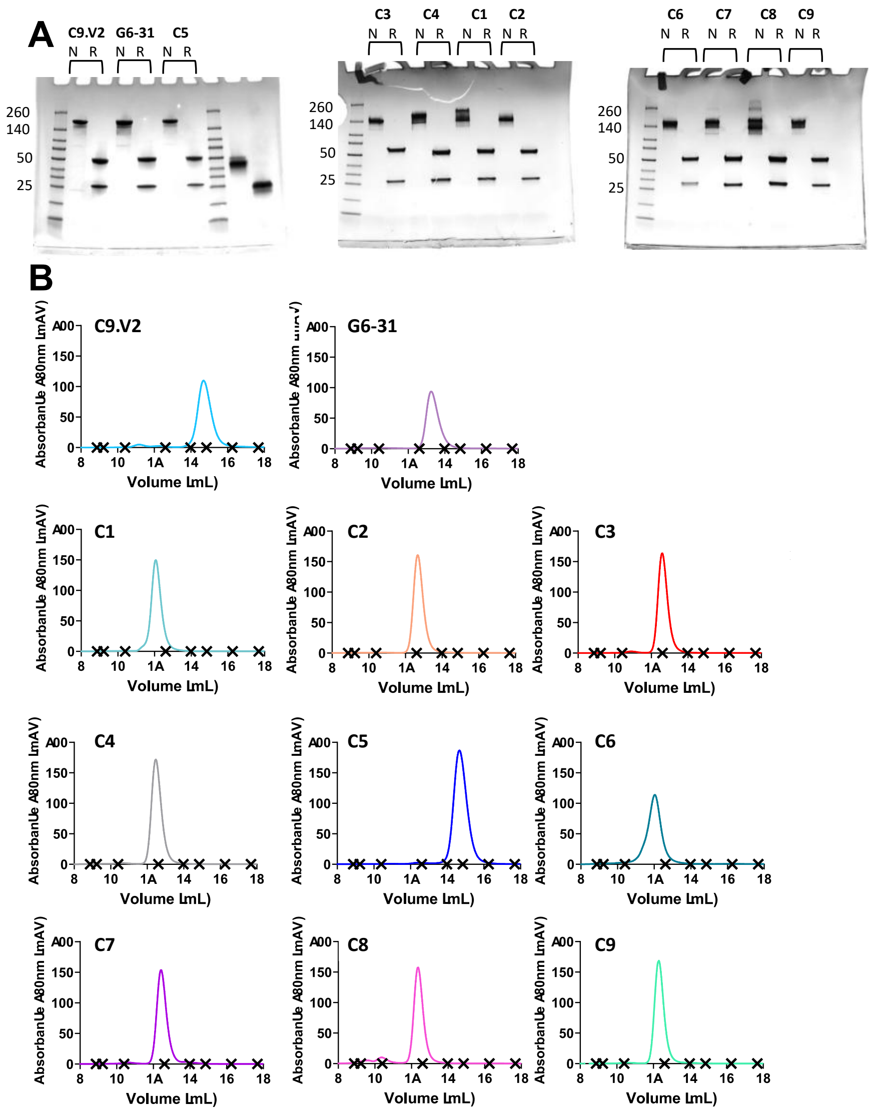

Figure S4. Antibody purity and monodispersity. (**A**), SDS-PAGE of purified antibodies with human IgG1/kappa domains. (**B**), Size exclusion chromatography of on Superdex S200. 'X' icons indicate the observed elution volume for the following standard markers: (8.78 mL = 2000 kDa, 9.33 mL = 669 kDa, 10.55 mL = 440 kDa, 12.62 mL = 150 kDa, 14.02 mL =75 kDa, 14.89 mL = 44 kDa, 16.88 mL = 29 kDa, 17.7 mL =13.7 kDa).

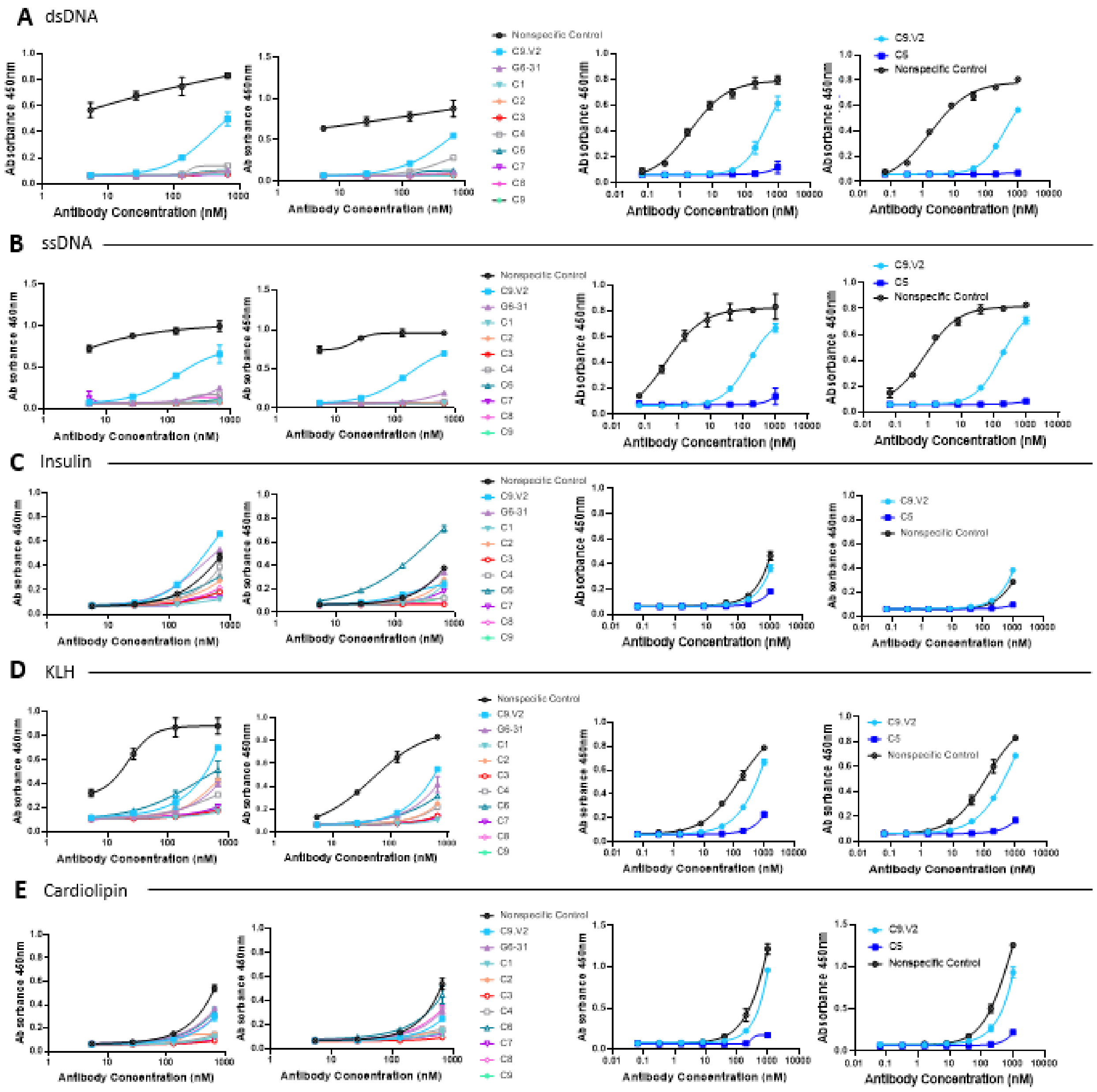

Figure S5. Discovered antibodies do not exhibit polyspecific reactivity. Supplemental poly-specificity ELISAs (**A**) against double stranded DNA, (**B**) single stranded DNA, (**C**) insulin, (**D**) keyhole limpet hemocyanin from *Megathura crentulata* (KLH), and (**E**) cardiolipin. Duplicate ELISAs shown for each comparison; data shown are the mean and range of technical duplicates.

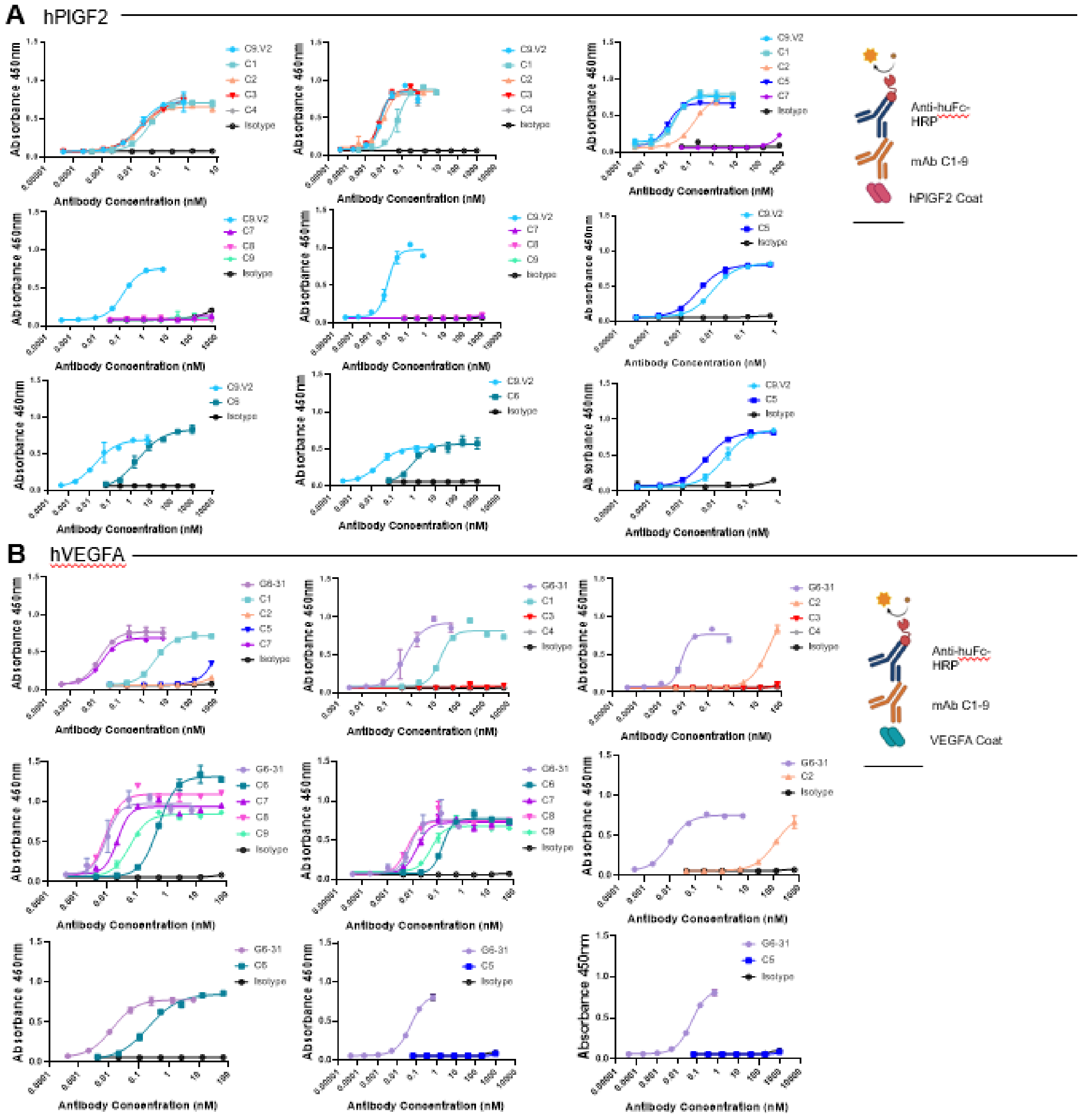

Figure S6. ELISA data demonstrating PlGF2 and VEGFA$_{165}$ binding profiles. Replicate data shown for all antibodies binding to plates coated with (**A**) PlGF2, (**B**) VEGFA$_{165}$. Duplicate ELISAs shown for each comparison; data shown are the mean and range of technical duplicates.

A hPIGF2

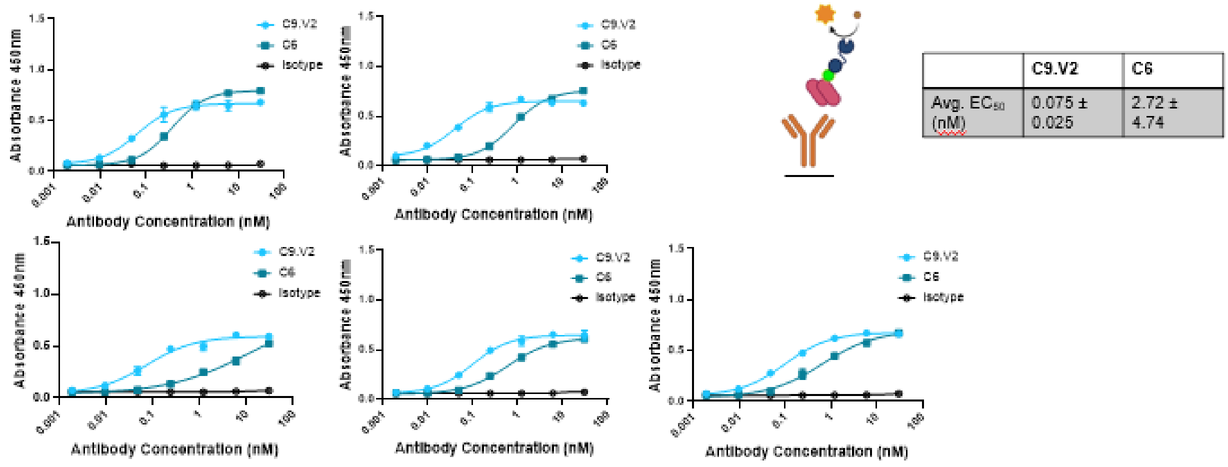

	C9.V2	C6
Avg. EC_{50} (nM)	0.075 ± 0.025	2.72 ± 4.74

B hVEGFA

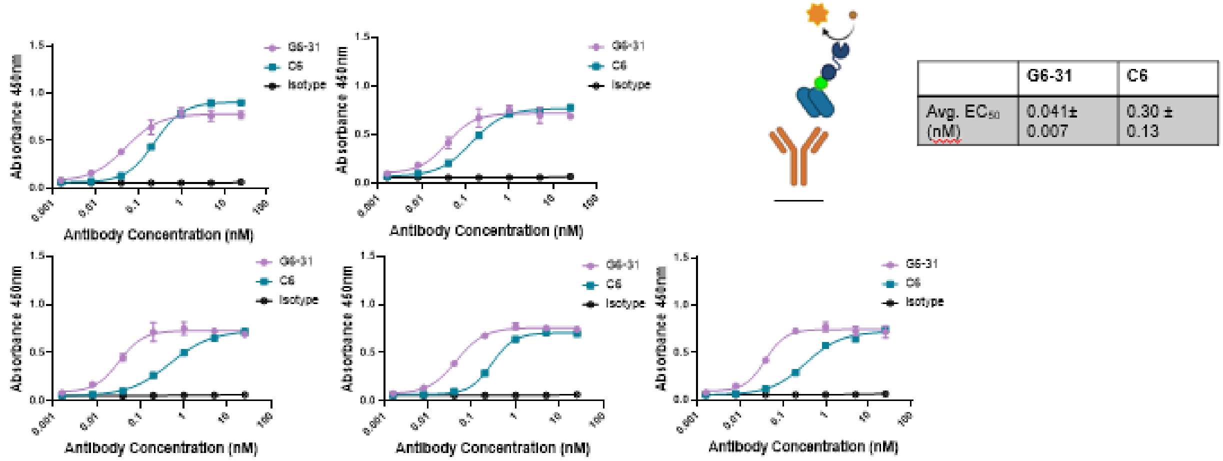

	G6-31	C6
Avg. EC_{50} (nM)	0.041± 0.007	0.30 ± 0.13

Figure S7. ELISA data demonstrates PIGF2 and $VEGFA_{165}$ binding profiles of cross-reactive antibody C6. These ELISAs were completed in a flipped orientation from **Fig. S6**, where antibody was used to coat plate and growth factor was biotinylated and detected with streptavidin-HRP. Replicate data shown for all antibodies binding to plates coated with (**A**) PIGF2, (**B**) $VEGFA_{165}$. Duplicate ELISAs shown for each comparison; data shown are the mean and range of technical duplicates.

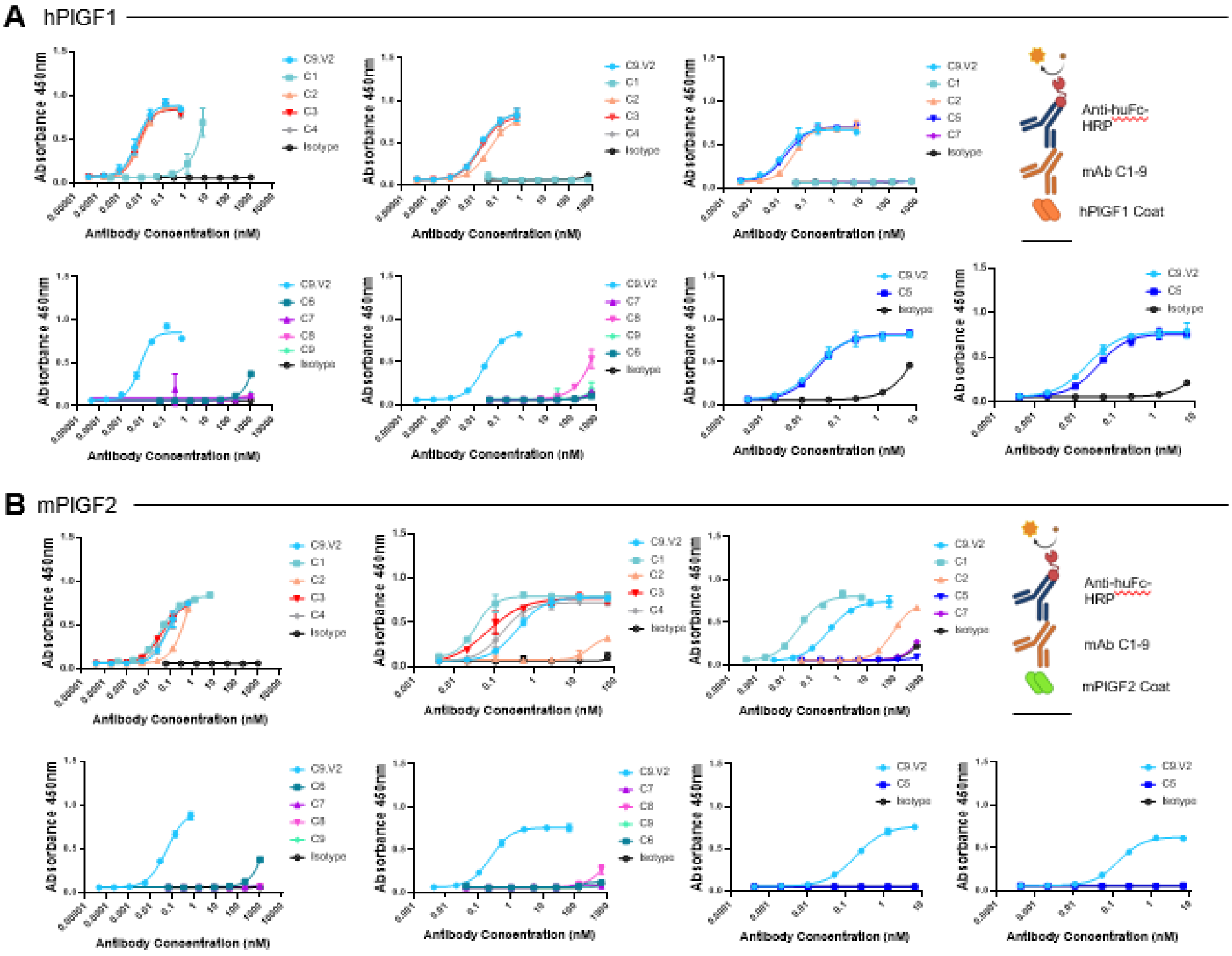

Figure S8. ELISA data demonstrating PlGF1 and mPlGF2 binding profiles. Replicate data shown for all antibodies binding to plates coated with (**A**) PlGF1, (**B**) mPlGF2. Duplicate ELISAs shown for each comparison; data shown are the mean and range of technical duplicates.

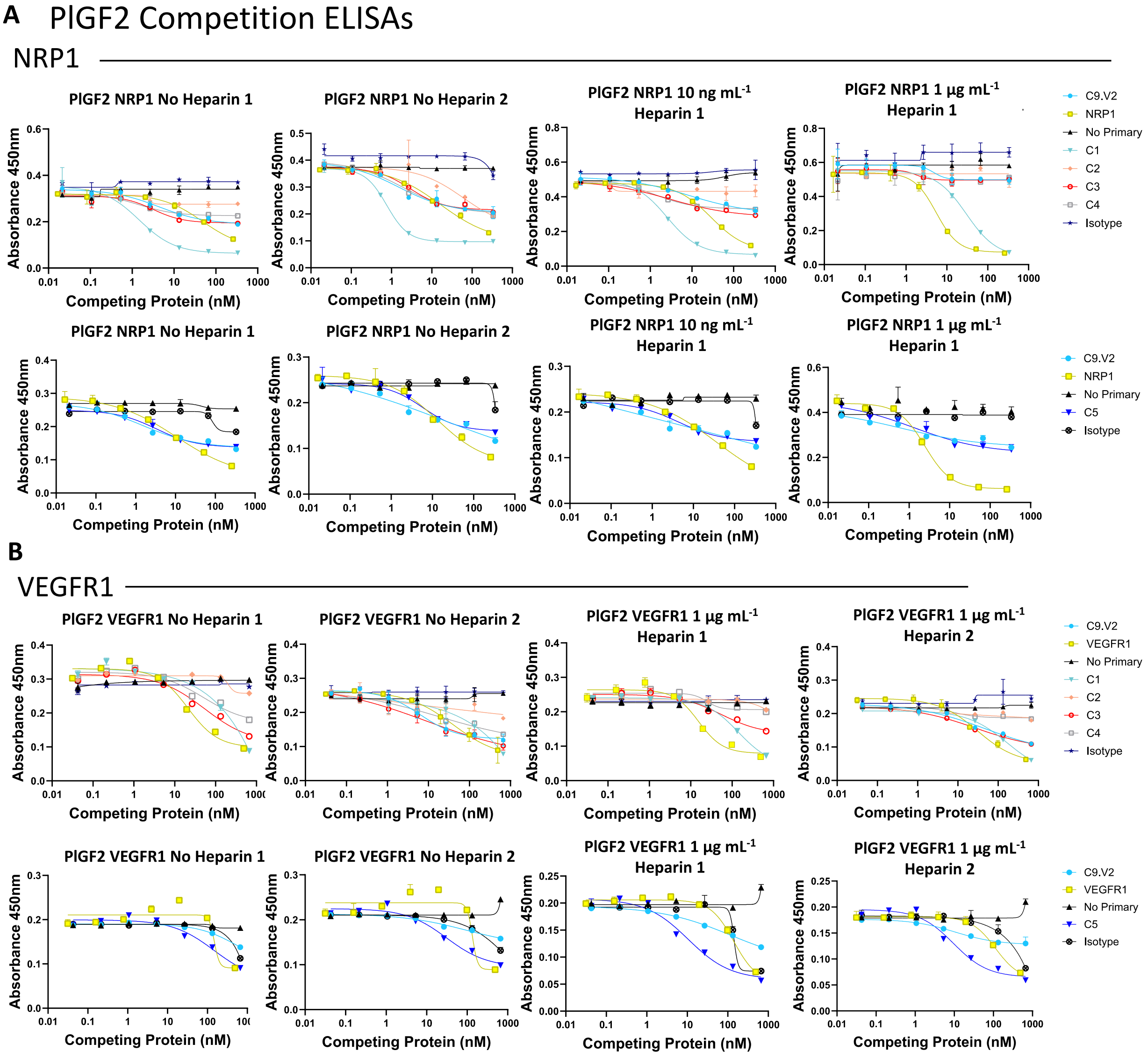

Figure S9. PlGF2 competition ELISAs. (**A**) Antibodies versus NRP1 in duplicate and (**B**) antibodies versus VEGFR1. Data shown are the mean and range of technical duplicates.

A VEGFA Competition ELISAs

NRP1

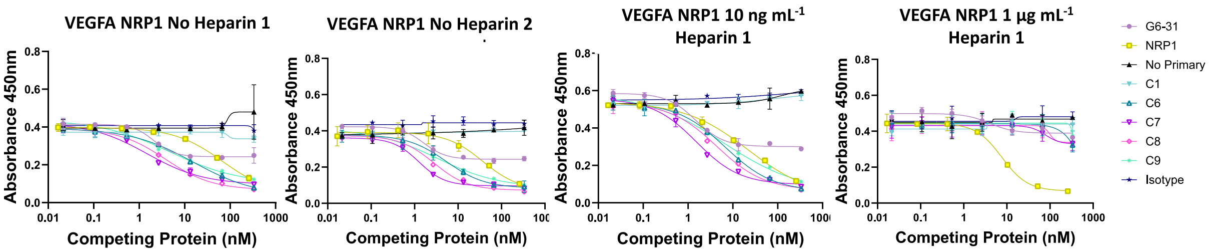

B

VEGFR1

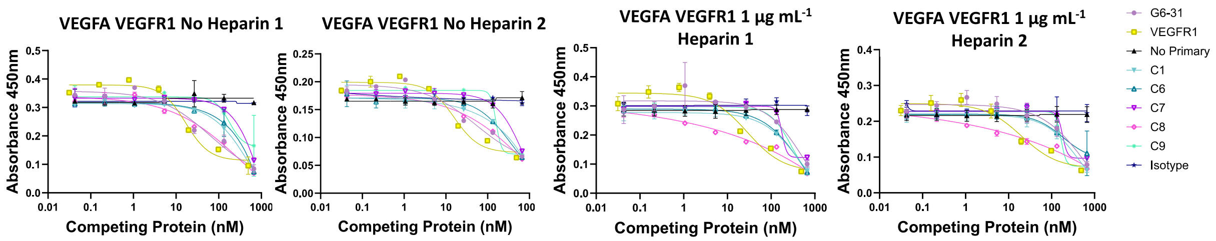

C Pertinent IC50 values

IC50s of NRP1 Competition without heparin

PIGF2	NRP1	C9.V2	C1	C5
IC50 (nM)	22.2 ± 17.6	6.5 ± 8.2	1.1 ± 0.5	4.5 1.7

VEGFA	NRP1	G6-31	C6	C7	C8	C9
IC50 (nM)	52.8 ± 22.1	1.9 ± 0.3	10.3 ± 7.0	1.4 ± 0.2	3.3 ± 0.7	4.1 ± 1.0

IC50s of VEGFR1 Competition without heparin

PIGF2	VEGFR1	C9.V2	C1	C5
IC50 (nM)	141.7 ± 10.0	307.7 ± 304.1	> 1000	89.7 ± 82.9

VEGFA	VEGFR1	G6-31	C6	C7	C8	C9
IC50 (nM)	16.3 ± 0.7	58.2 ± 6.9	> 1000	975.7 ± 284.7	643.2 ± 440.8	168.2 ± 12.8

IC50s of VEGFR1 Competition with 1 µg/mL heparin

PIGF2	VEGFR1	C9.V2	C1	C5
IC50 (nM)	75.1 ± 61.3	59.8 ± 55.9	155 ± 19.8	10.0 ± 0.7

Figure S10. VEGFA$_{165}$/ receptor competition ELISAs. (**A**) Antibodies versus NRP1 in duplicate and (**B**) antibodies versus VEGFR1. Data shown are the mean and range of technical duplicates. (**C**) Pertinent IC$_{50}$ values from NRP1 and VEGFR1 competition for PlGF2 and VEGFA$_{165}$.

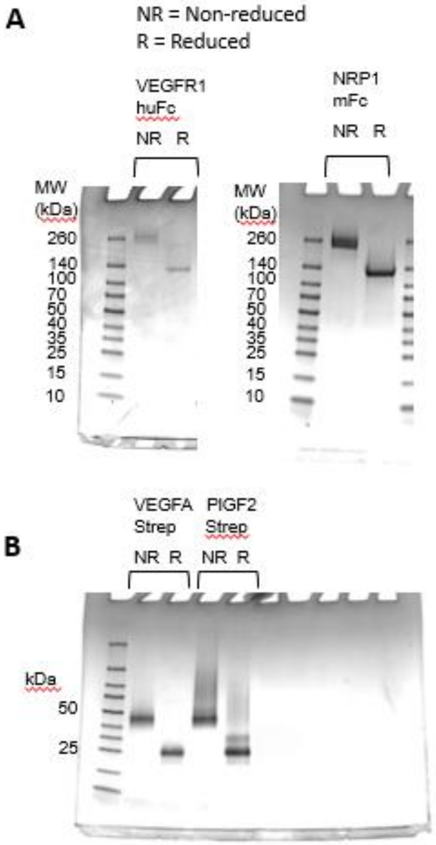

Figure S11. Production of recombinant receptors and growth factors. (**A**) SDS-PAGE of VEGFR1-huFc (203.6 kDa non-reduced, 101.8 kDa reduced) and NRP1-mFc (193 kDa non-reduced, 96.5 kDa reduced). (**B**) SDS-PAGE of VEGFA-2xStrep tagged (43.6 kDa non-reduced, 21.8 reduced) and PlGF2-2xStrep tagged (37.2 kDa non-reduced, 18.6 kDa reduced), purified by Streptactin resin.

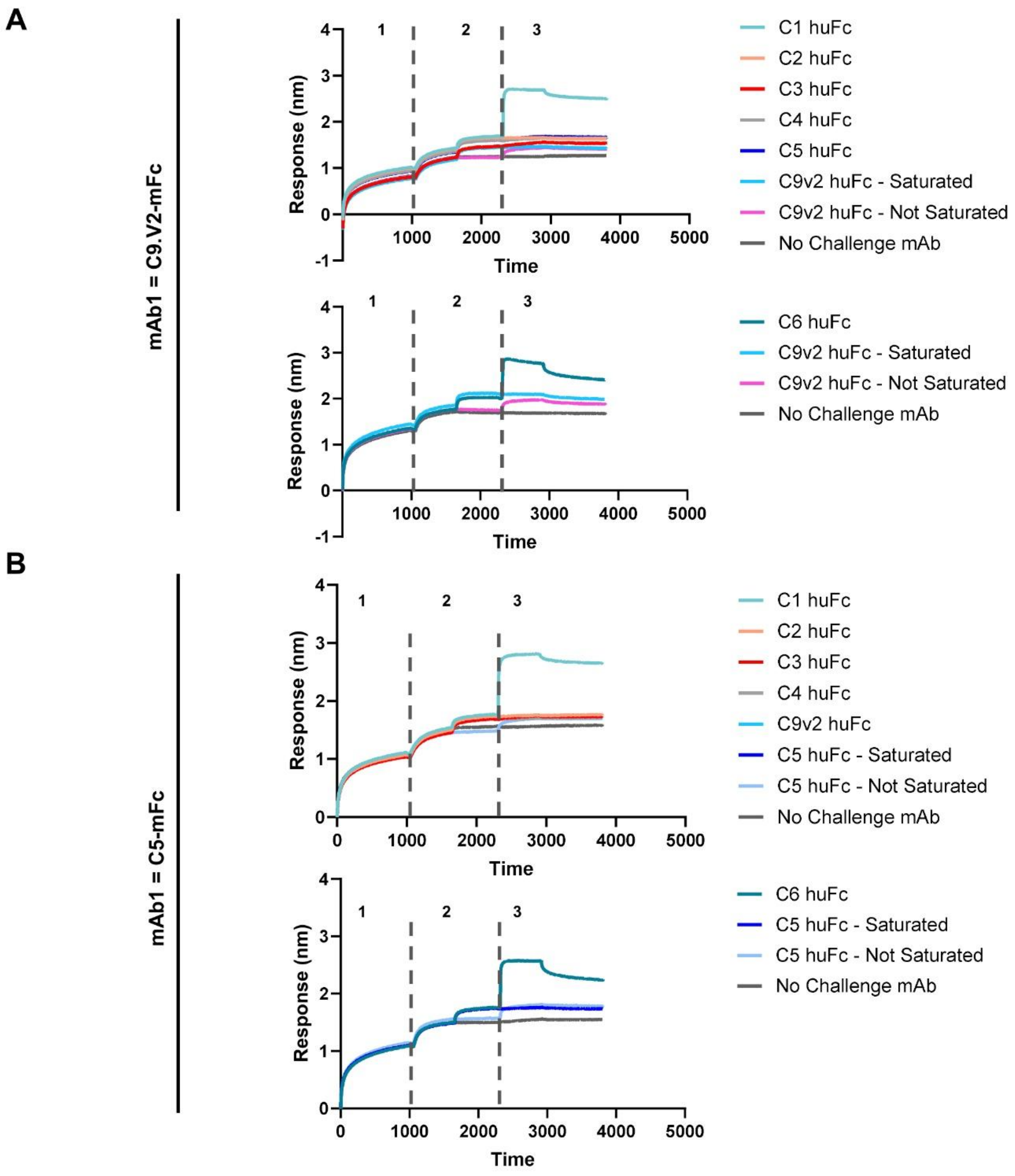

Figure S12. Antibody Epitope Binning against PlGF2. Representative BLI responses used to bin anti-PlGF antibodies into epitope groups using (**A**) C9.V2 or (**B**) C5 as the capture antibody. BLI traces displayed together were run simultaneously. Values shown in main Fig. 4 were calculated by subtracting the signal at 500s from the signal at 0s of association (step 3) and averaged across biological replicates.

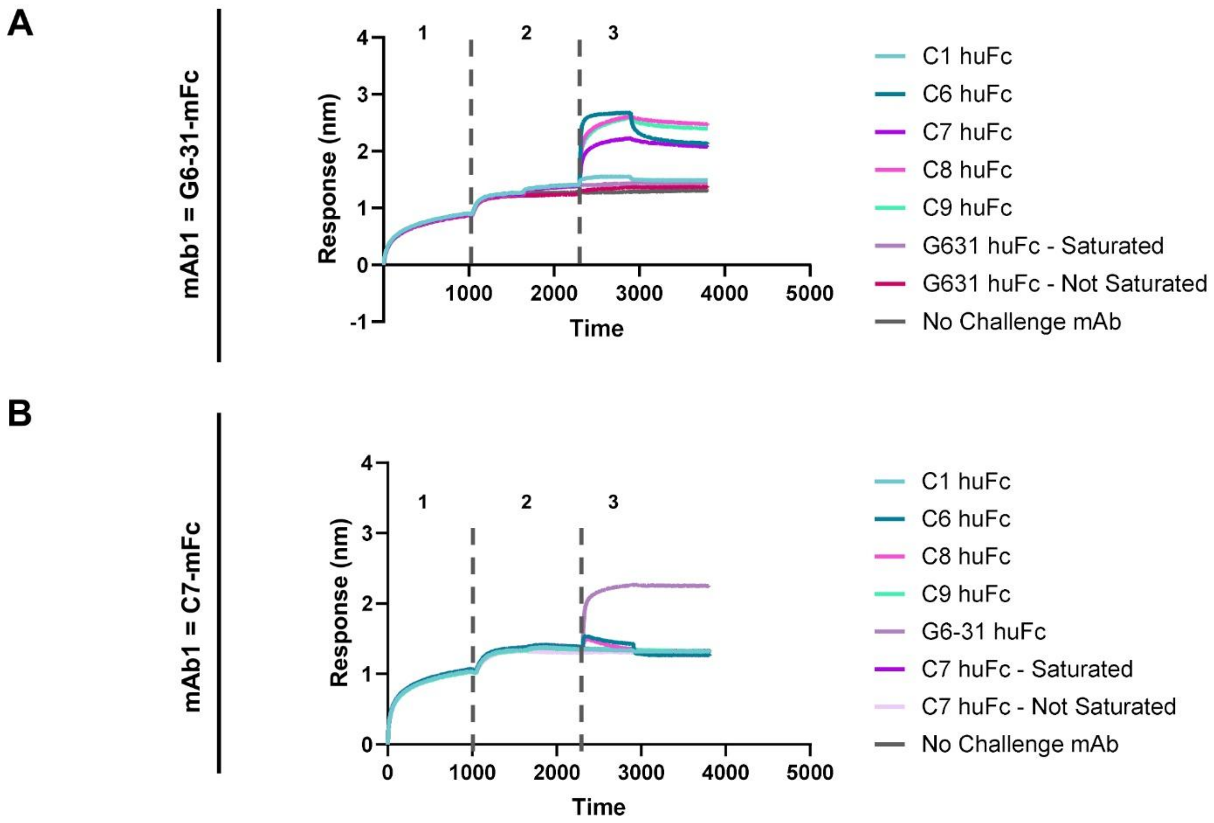

Figure S13. Antibody Epitope Binning against VEGFA$_{165}$. Representative BLI responses used to bin anti-VEGF antibodies into epitope groups using (**A**) G631 or (**B**) C7 as the capture antibody. BLI traces displayed together were run simultaneously. Values shown in main Fig. 4 were calculated by subtracting the signal at 500s from the signal at 0s of association (step 3) and averaged across biological replicates.

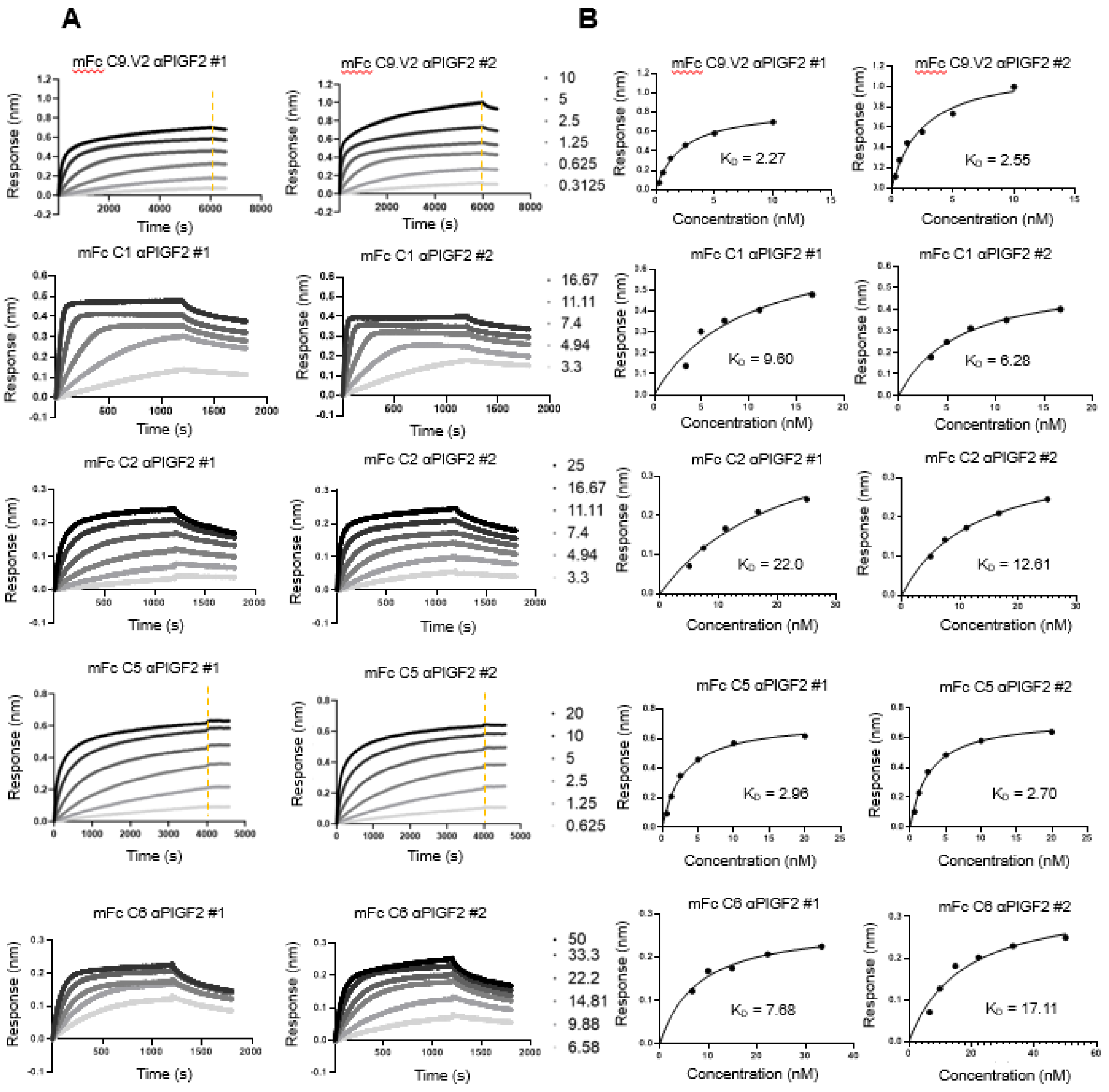

Figure S14. Anti-PIGF2 clones exhibit strong bivalent affinities for PIGF2. (**A**) BLI sensorgrams showing antibody binding to immobilized PIGF2, with technical duplicates. (**B**) The last ten association datapoints per dilution averaged and then fit to a one-site Langmuir isotherm to estimate effective K_D values. Reported K_D values are the range of two experimental replicates shown, each run with five concentrations in duplicate, and their averaged R^2 fit to a Langmuir isotherm.

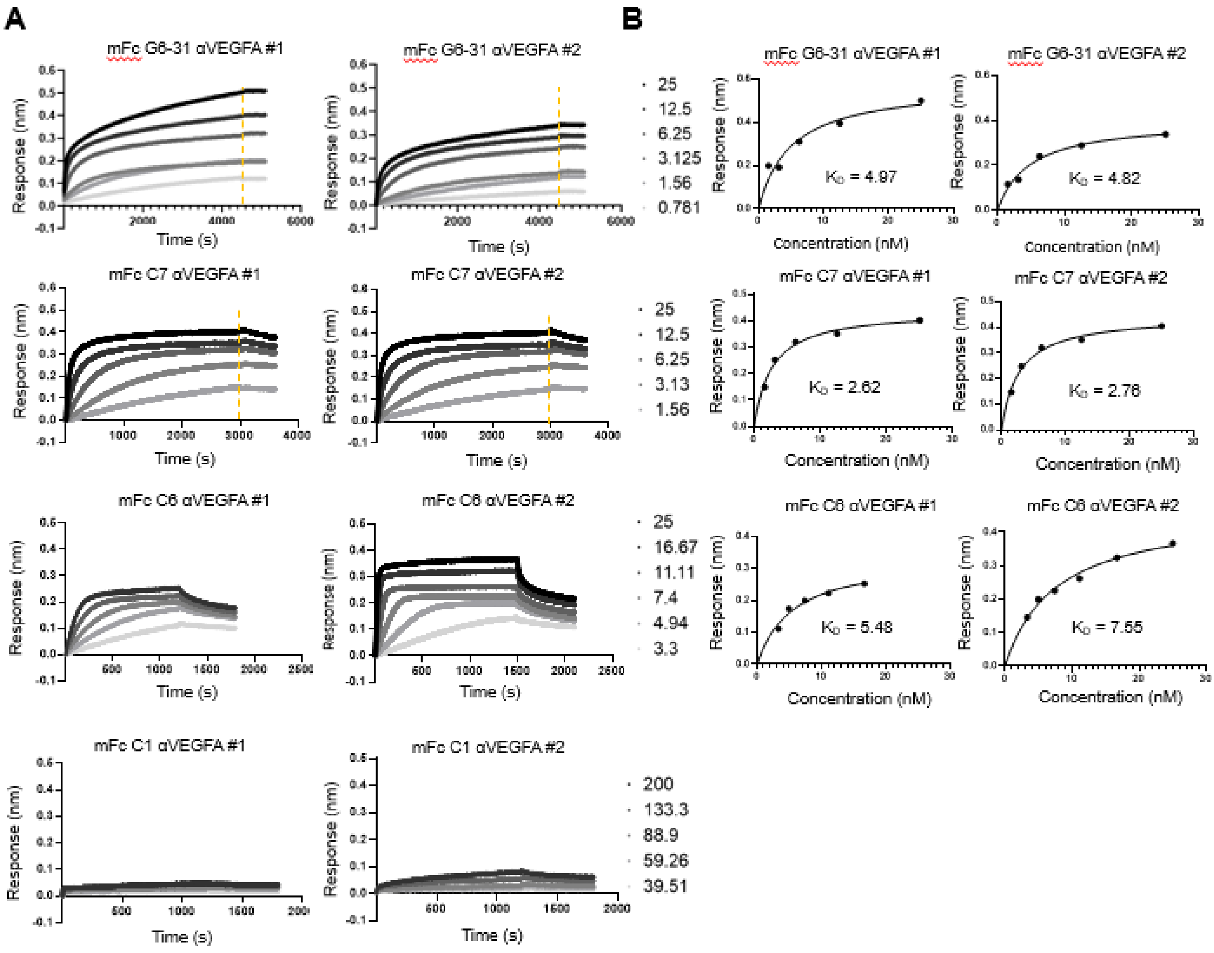

Figure S15. Anti-VEGFA clones exhibit strong bivalent affinities for VEGFA. (**A**) BLI sensorgrams showing antibody binding to immobilized $VEGFA_{165}$, with technical duplicates. (**B**) The last ten association datapoints per dilution averaged and then fit to a one-site Langmuir isotherm. Reported K_D values are the range of two experimental replicates shown, each run with five concentrations in duplicate, and their averaged R^2 fit to a Langmuir isotherm.

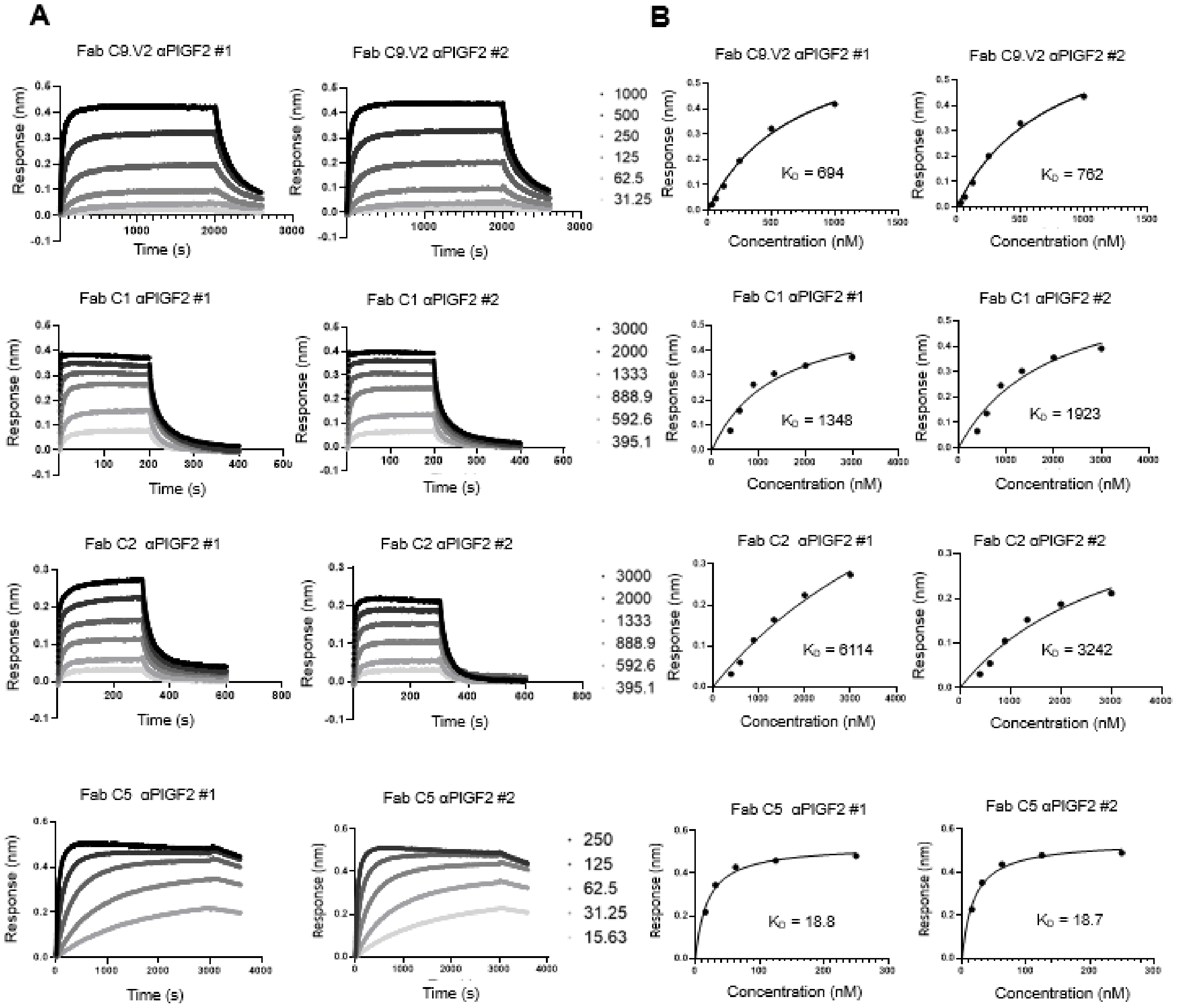

Figure S16. Antibody C5 exhibits strong binding to PlGF2 as a Fab. (**A**) Biolayer interferometry association and dissociation of Fabs to PlGF2, in duplicate. (**B**) The last ten association datapoints per dilution averaged and then fit to a one-site Langmuir isotherm. Reported K_D values are the range of two experimental replicates shown, each run with five concentrations in duplicate, and their averaged R^2 fit to a Langmuir isotherm.

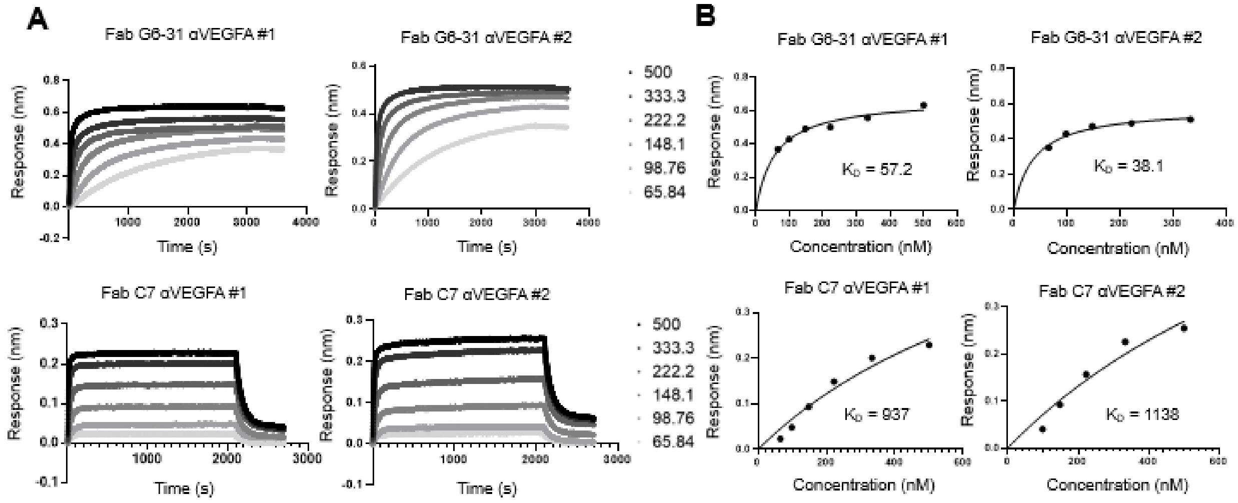

Figure S17. G6-31 and C7 exhibit strong or weak Fab-binding to VEGFA, respectively. (**A**) Biolayer interferometry association and dissociation of Fabs to $VEGFA_{165}$, in duplicate. (**B**) The last ten association datapoints per dilution averaged and then fit to a one-site Langmuir isotherm. Reported K_D values are the range of two experimental replicates shown, each run with five concentrations in duplicate, and their averaged R^2 fit to a Langmuir isotherm.

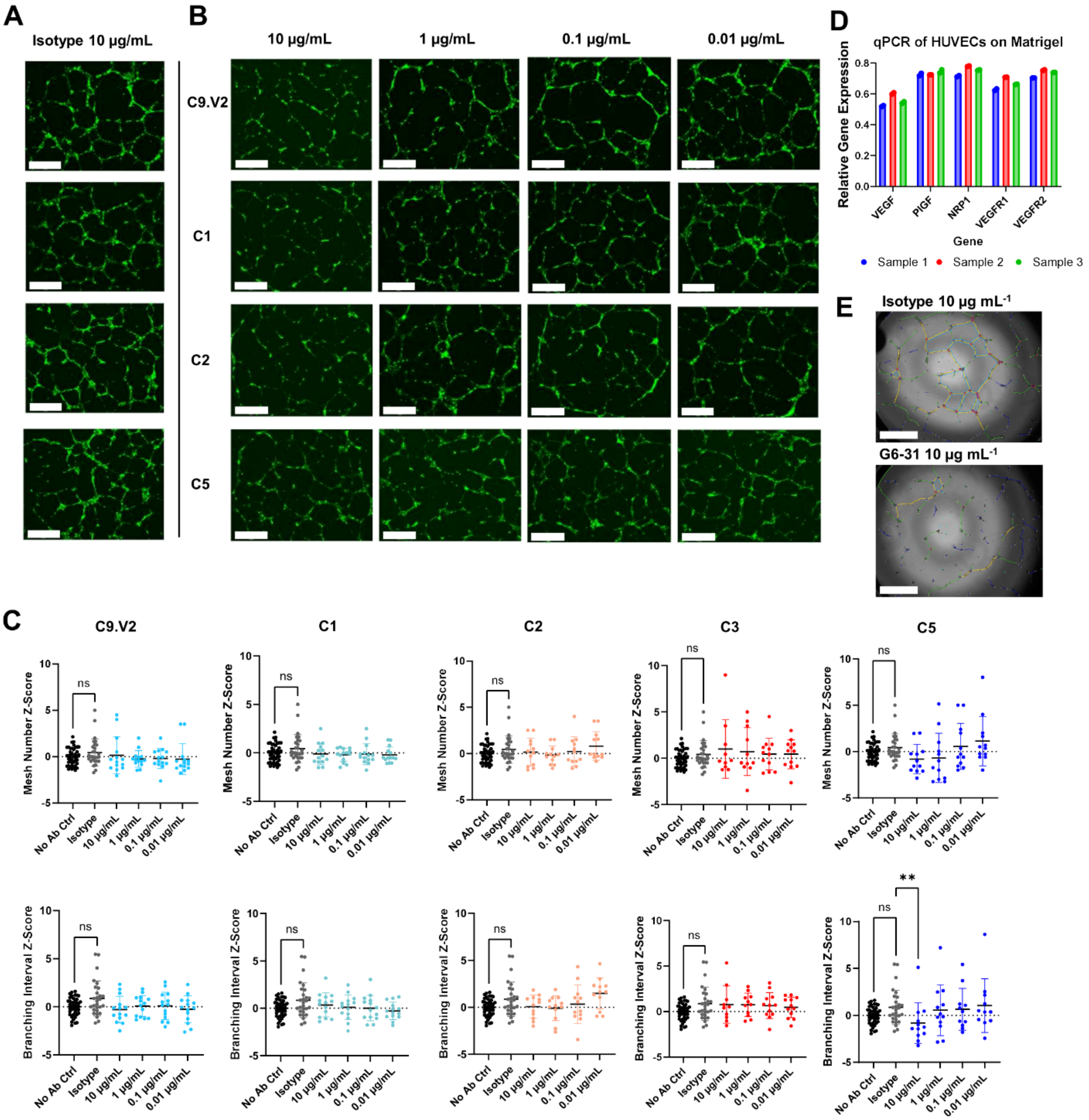

Figure S18. Anti-PlGF antibodies have no effect on HUVEC tube formation. GFP-fluorescent images of HUVEC tube formation depict the isotype dosed at 10 μg/mL (**A**) and the PlGF Ctrl, C1, C2, and C5 dosed at 10, 1, 0.1, and 0.01 μg/mL (**B**). (**C**) Angiogenesis Analyzer ImageJ Plugin measurements of Mesh Number and Branching interval are negligibly different between anti-PlGF antibody conditions and the isotype control. (**D**) Relative qPCR gene expression of VEGF, PlGF, NRP1, VEGFR1, and VEGFR2 derived from three sets of 16-pooled HUVEC tube formation replicates. (**E**) Example of the Angiogenesis Analyzer ImageJ plugin measuring tube formation for isotype- and G6-31-treated cells.

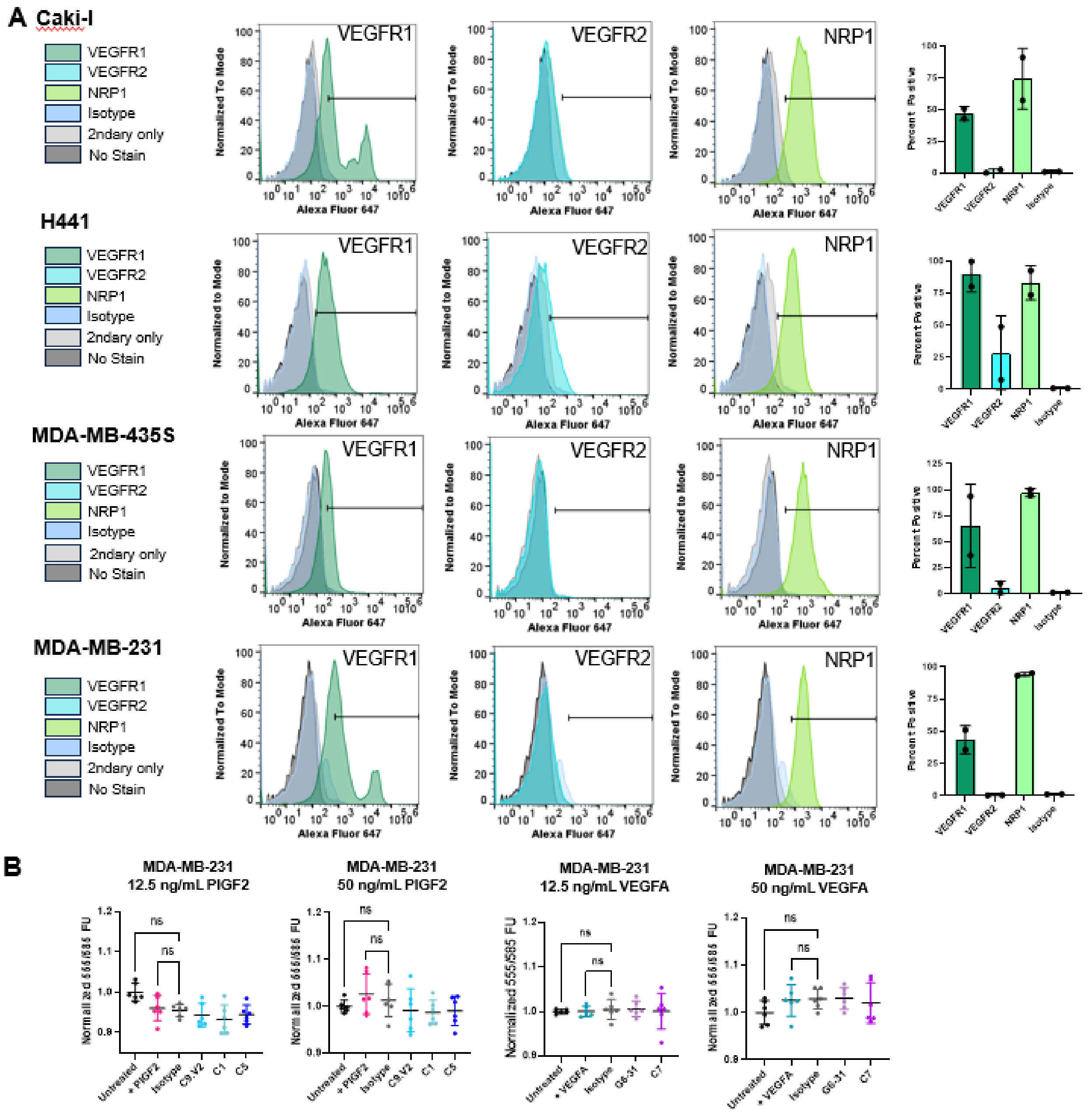

Figure S19. All cancer cells evaluated express VEGFR1 and NRP1. (**A**) Flow cytometry and percent positive of Caki-I, H441, MDA-MB-435S, and MDA-MB-231 cancer lines stained for VEGFR1, VEGFR2, and NRP1. Percent positive was defined by ~1% overlap with the isotype control staining. (**B**) Metabolic Inhibition Assay with MDA-MB-231 cells which demonstrates cells were not stimulated by PlGF2 or $VEGFA_{165}$ at 12.5 or 50 ng/mL after starving in serum-free media.

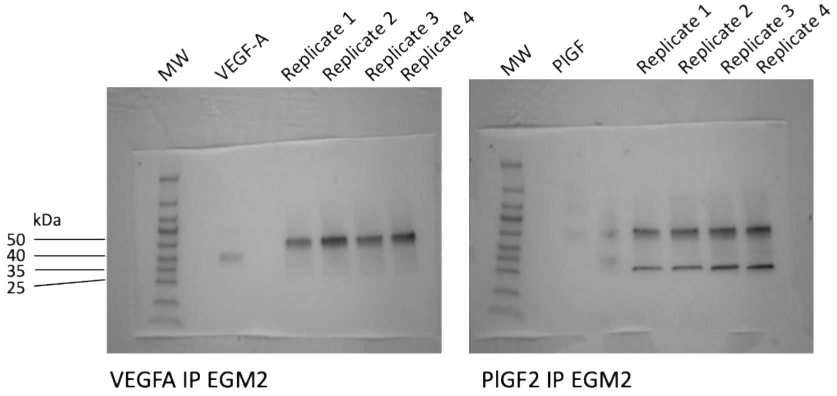

Figure S20. Immunoprecipitation of HUVEC-conditioned media after 24 hrs of cells growth using EGM2; with unconditioned media and three replicates shown.
